# ‘They’re born to get breastfed’- how fathers view breastfeeding: a mixed method study

**DOI:** 10.1186/s12884-018-1827-9

**Published:** 2018-06-18

**Authors:** Emily Hansen, Leigh Tesch, Jennifer Ayton

**Affiliations:** 1 School of Social Sciences, University of Tasmania, Private bag 22, Hobart, TAS 7001 Australia; 2 Public Health, College of Health and Medicine, University of Tasmania, Private Bag 135, Hobart, TAS 7001 Australia

## Abstract

**Background:**

Fathers’ attitudes and actions can positively or negatively affect mothers’ intentions to breastfeed, breastfeeding duration and exclusivity. In-depth information about fathers’ perspectives on breastfeeding are largely absent in the literature about infant feeding. The objective of this research was to investigate how fathers view breastfeeding.

**Methods:**

This mixed method study recruited Tasmanian fathers with children < 24 months of age. Fathers completed a questionnaire and participated in either semi structured one-on-one or group interviews. Transcripts were analysed using a process of iterative thematic analysis.

**Results:**

Twenty-six fathers participated in the study. They had a mean age of 34 years and just over half were first time fathers. A total of 13 fathers lived in areas classified by SEIFA as disadvantaged. Twenty-one reported they had decided as a couple to breastfeed their current child. Fathers’ views on breastfeeding are complex, multi-layered and change over time: as babies get older, as fathers get more familiar with feeding babies, when feeding practices change and when family circumstances change. Four thematic categories related to how fathers view breastfeeding were identified; Breastfeeding as healthy and natural, the value of breast feeding and breastmilk, a pragmatic approach to breastfeeding and Breastfeeding as something achieved or imposed.

**Conclusion:**

Fathers in our study valued breastfeeding and saw it as healthy and natural for babies. However, many of the fathers in our study had seen their partners struggle with breastfeeding. As a result some also viewed breastfeeding as a potentially harmful practice for mothers. Their accounts demonstrated that breastfeeding problems affect families, not just mothers and infants. There is scope for improvement in the care of women during and after birth to reduce breastfeeding problems and for fathers to learn more about breastfeeding prior to the birth of their child.

**Electronic supplementary material:**

The online version of this article (10.1186/s12884-018-1827-9) contains supplementary material, which is available to authorized users.

## Background

Breastfeeding makes an important contribution to both child and maternal health and wellbeing [1]. Exclusive breastfeeding (where the child receives only breastmilk) to six months has a demonstrated positive impact on health during infancy that appears to continue into adulthood [2–4]. In high-income countries such as Australia low rates of breastfeeding are generally associated with social disadvantage [5, 6]. However, even among mothers from advantaged backgrounds, exclusive breastfeeding rates are lower than those recommended in the World Health Organization (WHO) guidelines [7]. The WHO recommends that babies be exclusively breastfed till six months of age, and then for breastfeeding to continue alongside suitable complementary foods for up to two years and beyond [8].

Fathers’ views on breastfeeding are powerful determinants of mothers’ infant feeding decisions and practices. Fathers’ attitudes and actions can positively or negatively affect mothers’ intentions to breastfeed, breastfeeding duration and exclusivity [9–12]. Some studies suggest that fathers’ perceived attitudes may be among the most powerful influences shaping mothers’ feeding choice and whether or not the mother initiates and then continues to breastfeed [5, 7].

Much of the work about fathers and breastfeeding has focused on how mothers describe the role or importance of fathers. Many, but not all of the limited number of existing studies have used questionnaires to explore fathers’ attitudes and knowledge about breastfeeding [13–16]. Fathers appear to hold complex and sometimes contradictory views on breastfeeding. A number of studies have found that most fathers hold positive views towards breastfeeding and would like their child to be breastfed or at least to receive breastmilk [9, 15, 17, 18]. However, some fathers also describe feeling unprepared for breastfeeding and left out or unimportant due to limited opportunities to actively feed their babies and the close emotional ties between mother and infant [19, 20]. Fathers whose partners have experienced breastfeeding difficulties describe breastfeeding as something that can be traumatic and emotionally fraught [21]. As with mothers, how fathers view breastfeeding and their actions related to breastfeeding appear to be shaped by their age, socio-economic status, life stage, ethnicity and social location [22]. Younger men and men who are about to become fathers for the first time often report that breastfeeding is not something they know much about or have given much thought to [16, 23].

There is a body of sociological scholarship focused on changing expectations about fatherhood in Western societies since the 1970s [24]. There is considerable debate within this literature about the extent to which changes in ideas about fatherhood and parenting are reflected in the actual division of labour within families, but agreement that contemporary Western ideas about parenting have changed [25, 26]. The contemporary father is now expected to be present physically and emotionally throughout the pregnancy, birth and child rearing process [27]. Thus, the ways that fathers view breastfeeding and infant feeding is important not only in terms of how fathers might shape the actions and views of mothers but also because fathers themselves are more likely to be actively involved in feeding babies and young children. A desire to be an involved co-parent may pose challenges for fathers whose partners exclusively breastfeed unless they are able to recognise that they make a valuable parenting contribution by providing support to breastfeeding mothers, while also identifying ways to nurture and feel physically close to their infants that do not involve feeding [19]. It has been suggested that while emotional support from fathers promotes breastfeeding, fathers who spend time caring for babies and/or who actively co-parent may be detrimental to breastfeeding due to a higher incidence of bottle feeding in these families [10].

Fathers’ opinions are important when mothers make the decision about how to feed their babies and their support for breastfeeding extends breastfeeding duration [7]. However, fathers’ own perspectives on breastfeeding are largely absent in the literature about infant feeding. This article helps to address this evidence gap by focusing on the experiences and views of Australian fathers who have infants or young children aged under 24 months.

## Methods

### Design, aims and setting

The mixed method study was conducted in Tasmania, Australian between March–December 2013, and has been reported elsewhere [28]. The research was conducted by three female researchers: a senior health sociologist, a midwife and an occupational therapist. The research aimed to explore and describe how Tasmanian fathers of children aged 0–24 months see their role in infant feeding, their experiences of infant feeding and their views on breastfeeding. This article presents only those findings relevant to how fathers viewed breastfeeding.

### Ethics

Ethical approval for the study was granted by the Human Research Ethics Committee (Tasmanian) Network (approval number H0011838). All participants gave free and informed written consent prior to any collection of data.

### Sample and recruitment

Our purposeful sampling strategy was to recruit fathers of different ages living in different regions of Tasmania with children aged under 24 months of age with at least half of the sample living in areas characterized by socioeconomic disadvantage based on the Socio-Economic Index for Areas index (SEIFA). The index is constructed by the ABS principally from 5 variables related to person, place and dwellings and is assigned to areas not to individuals [29]. The indexes are reported in quintiles with 1 equalling the most disadvantaged, and 5 equalling least disadvantaged are [29]. Study information inviting people to join the research was distributed via email, web sites, flyers and support services for parents. We also used snowballing techniques where fathers already recruited to the study were invited to pass information about the study to other men that they knew. Each participant received a $20.00 supermarket gift voucher in recognition of their time. Recruitment ended after 26 fathers had joined the study. We had used the time available to the research team for recruitment and were confident that we had enough quality interview data to support a meaningful analysis. We were also observing indications of data saturation. There were increasing numbers of similarities across interviews and a general slowing down of new codes/themes in the later interviews.

### Interviews and questionnaires

The three authors conducted semi structured interviews based on a list of open-ended questions based on an interview guide (Additional file 1). For example, “tell me a bit about yourself and your baby” and “tell me about your experiences of feeding your baby?” We conducted 17 father only interviews, 3 mother/father combined interviews, 1 group interview with two fathers and a second group interview with 4 fathers. The group interviews occurred when either a small pre-existing group of fathers or a father/mother dyad told us that they would prefer to be interviewed together. The fathers in the study seemed comfortable talking about breastfeeding with the female interviewers. Interviews were conducted in homes and community settings and were audio recorded. Field notes were written after some interviews. Prior to the commencement of an interview each participant completed a demographic questionnaire. The questionnaire included Australian Bureau of Statistics (ABS) standardised measurements for socio-economic status, educational standard attained, ethnicity and employment status. The questionnaires also collected data about age of the father, mother and youngest child, the child’s gestation at birth, parity, place of birth (hospital private/public; home), the intended method of feeding prior to this child’s birth and the current method of feeding (last 24 h). All infant feeding terms are consistent with the WHO Indicators for assessing infant and young child feeding practices [4].

### Data analysis

Interview audio-recordings were fully transcribed as soon as possible and any field notes and summaries of questionnaire data were attached to the relevant transcripts. Transcripts were identified using a pseudonym. They were analysed using a process of iterative thematic analysis that utilized coding and the constant comparison technique [30]. Each member of the research team read and re-read hard copies of the transcripts and then either coded in pen directly onto the transcripts, later transferring their memos and key examples to word documents, or used the software program NVIVO to record coding and memos. Transcripts underwent an initial analysis soon after the interview or focus group was conducted so the researchers could take insights from that interview into the subsequent interviews [31]. The research team held a series of group analysis meetings where we engaged in a process of reflexive discussion, comparing our coding and refining the analysis. The first author then regrouped the codes and initial themes into larger thematic categories that were linked to the research questions. The other two researchers then reviewed and approved the final thematic categories, thus completing the analysis. Data collected from the questionnaires was cleaned and frequencies, distributions and standard deviations from the mean were generated using the statistical program STATA Version [32].

## Results

Twenty-six fathers participated in the study. They had a mean age of 34 years and just over half (14 fathers) were first time fathers. A total of 13 fathers lived in areas classified by postcode as either SEIFA quintile 1 (11 fathers) or 2 (2 fathers) both of which can be considered ‘disadvantaged’. The majority (24 fathers) were living with the mother of their child [either married or de facto]. Fourteen fathers reported that their partner had breastfed before (some mothers had children with their previous partner) and 21 reported they had decided as a couple to breastfeed their current child. At the time of the interview or focus group, 16 of the babies were eating other foods and milks. The mean age of the children at the time of the interview/focus group was 18.2 months. Ten children were receiving breastmilk as part of their regular diet (includes any breastmilk either directly from the breast, and or expressed and other foods and fluids).

Overall, our analysis of the interview data found that fathers’ views on breastfeeding are complex, multi-layered and change over time: as babies get older, as fathers get more familiar with feeding babies, when feeding practices change and when family circumstances change (for example when mothers return to work). Fathers told us that they had not thought much about breastfeeding until their first child was born and then it became a big issue in their family at certain points in time. The first of these was during the initiation and establishment of breastfeeding when breastfeeding problems were most common. The other key times were, if breastfeeding was associated with babies waking in the night and at weaning. Four thematic categories related to how fathers view breastfeeding were identified; breastfeeding as healthy and natural, the value of breast feeding and breastmilk, a pragmatic approach to breastfeeding, and breastfeeding as something achieved or imposed.

### Breastfeeding as healthy and natural

Breastfeeding was described as healthy, natural, promoting bonding between mother and child, cheaper than using formula, convenient and the best option if you can do it:

I think it’s important, getting the child close to their mother and obviously breastmilk is, obviously all the good stuff in there, so it’s obviously good to get that into their system as well. But I think it’s a good bond between mum and child. (William)

It’s good for kids when they get breastfed. It’s good and helps them with their development. … they’re born to get breastfed… A natural way of growing up. (Otto)

Fathers, while aware that breastfeeding had health benefits for babies, seemed less aware of exclusive breastfeeding or the links between exclusive breastfeeding and health outcomes. Many described supplementing babies under six months of age with formula or cereal. Some fathers, particularly those whose babies had been fed mainly with formula or whose babies had only been breastfed for a short period, talked about breastfeeding being important for young babies but less so for older babies:

Interviewer: So how did you make that decision? (to stop breastfeeding)

Father: … it just seemed like the right time because he was getting big enough and he got teeth very early, and it was just so much strain on my wife and just the drain of using the milk all the time… (Paulo)

Fathers were aware that breastfeeding is beneficial for mothers in terms of bonding with their child. However, no fathers discussed health benefits for mothers that arise from breastfeeding. In fact, for some men whose partners had stopped breastfeeding prematurely or who had chosen never to breastfeed, breastfeeding was viewed as being a potential or actual threat to a mother’s or child’s health and wellbeing due to tiredness and stress associated with trying to breastfeed.

### The value of breastmilk

Some of the fathers in our study valued breastfeeding and/or breastmilk highly and worked very hard with the mothers of their child to enable their child to be breastfed or fed with expressed breastmilk (EBM). For example, one family in the study recruited several friends to form an informal milk bank in order to supplement their own low supply of breastmilk:

We actually got talking to our friends basically and then they said, or three of them said they would express extra milk and give it to us to give to (the baby). So even though we’re stuffing around with formula and he’s not getting fully breastfed, he’s getting about 50%. In the early stages, we were getting three people dropping off milk. … He’s nine months now so we’re still getting some from a friend as well. (Geoff)

Some fathers spoke about breastfeeding and the use of EBM as being equivalent. To some extent breastmilk was viewed as a product distinct from the process of breastfeeding. For those fathers whose children were breastfed, bottle feeding usually only happened occasionally, either to give the mother a break or to help out if the mother had to do something else. However, some families regularly fed their child with bottles of EBM and combined this with breastfeeding. In contrast, some other fathers of breastfed children enjoyed the experience of bottle feeding so much that they encouraged their partners to express milk so they (the father) could feed it to their child. Other fathers said that they worried about breastfeeding because it is not possible to see how much the child is getting to eat and advocated the use of EBM so that the quantity of milk could be assessed.

Fathers spoke about the desirability of breastfeeding and the health benefits of breastfeeding irrespective of whether or not their own children had been breastfed. We noted that fathers living in areas characterized by SEIFA as most disadvantaged (quintiles 1–2) were more inclined to describe how their partner had stopped breastfeeding before the child was six months old, and to say that men often feel uncomfortable when women breastfeed in public.

### A pragmatic approach to breastfeeding

Fathers valued a healthy happy mother and child more than breastfeeding. When they perceived that breastfeeding was detrimental to this desired outcome, they were accepting of the perceived need to stop breastfeeding and supplement breastfeeding with formula milks.

From our point of view, my world didn’t come crashing down because we didn’t breastfeed, but I was disappointed because (my wife) was disappointed. She really wanted that. She really was determined that it would be that way. (Cory)

It was hard first time. We are learning a lot now. We’ve just come to the conclusion that we know what’s best for (the baby) and everyone else can, not jump, but you know. Obviously, it’s (formula) safe, but we know what’s best and we’ve got our gut feelings. (Joel)

This meant that unlike many mothers, fathers did not describe feeling like a failure if breastfeeding was ceased prematurely due to breastfeeding problems or if the mother did not want to breastfeed. In some cases, infant formula and bottles were viewed as essentially saving the child’s or the mother’s health when breastfeeding had not worked out as planned. Fathers described feeling thankful that the bottle and formula were available as their child had needed them:

… at that point, we made the decision to go full formula which I thought was good. It took the pressure off. It allowed me to do the feeds as well, it took the pressure of her, she could get some sleep. We ended up being really lucky and getting a baby who straight away starting sleeping either with one feed or through the night, little angel child. … (Cory)

### Breastfeeding as something achieved or something imposed

Many fathers spoke about breastfeeding as something they and their partners had really wanted to do, and when obstacles occurred they had struggled to overcome them in order to achieve their goal of breastfeeding. For them, breastfeeding was something they had achieved through perseverance:

… I was keen to try and continue the breastfeeding and get it all sorted out, I didn’t want to just give up and go onto formula. Because in the end we didn’t really end up having to give her a lot of formula, it was only a couple of days and a couple of doses really. (Keith)

Conversely, for some fathers (and mothers) breastfeeding was not something they strove to achieve. Rather, it was experienced as a burden, something that other people encouraged them to do even when they considered that it was not right for them. These men associated breastfeeding with being told what to do, either while in hospital immediately after giving birth, or in the first few weeks or even months following the birth. For these fathers [many of whose children had been switched from breastfeeding to infant formula soon after leaving hospital], breastfeeding was something that, while good in principle, had in practice been experienced as a burden and imposition:

Ours were only done in hospital (breastfed). As soon as we came home they were stopped. (Martin)

… the nurses you take the babies to, they’ve got their own set of opinions and that’s it, and, they’re right and the parents are always wrong, especially with breastfeeding. (Chester)

If people don’t want to breastfeed that’s their choice, but I don’t think it should be forced on you. (Joel)

Discussion about feeling pressured to breastfeed from health professionals was common across many of the interviews.

## Discussion

Fathers’ infant feeding preferences are an important determinant of women’s infant feeding decisions and it is encouraging to see that the Tasmanian fathers in our study viewed breastfeeding as natural and healthy for their children. However, they connected many of the benefits of breastfeeding with the breastmilk and thus made little or no distinction between breastfeeding from the breast and the use of expressed breastmilk. Their acceptance of EBM as being equivalent to breastfeeding has implications for the duration of exclusive breastfeeding, as regular feeding with EBM has been shown to increase the risk of early cessation of exclusive breastfeeding [7]. This is particularly relevant if fathers encourage the use of EBM as a way for them to become involved in feeding young infants. The fathers in our study appear to be largely unaware of breastfeeding as something that carries health benefits that are associated with the method of feeding rather than just the milk. Nor were they aware of health benefits for mothers other than facilitating bonding with their child. In fact, several fathers viewed breastfeeding as something that could be harmful for mothers when breastfeeding was perceived as being imposed by health professionals against a mother’s wishes, viewed as providing insufficient food for their child, or had resulted in pain and or emotional distress for their partners.

It is apparent in our findings that fathers’ views on breastfeeding are shaped by their own experiences and observations. When these were positive, as was the case for some fathers in the study, breastfeeding was seen as important, desirable and contributing positively to the experience of being a parent. However, many of the fathers in our study had seen their partners struggle with breastfeeding. Their accounts demonstrated that breastfeeding problems affect families, not just mothers and infants. When breastfeeding was perceived as a threat to mother’s or child’s wellbeing the fathers in our study viewed the use of infant formula as comparable to breastmilk and preferable to ongoing attempts to breastfeed.

It appears that fathers would benefit from learning more about breastfeeding including information about the benefits of exclusive breastfeeding for infants and mothers, assessing milk supply; known strategies for facilitating breastfeeding [such as the importance of skin to skin contact] [33], and techniques to assess the wellbeing of their child so they can be reassured their child is getting enough food [34]. Education and services about infant feeding should be delivered at a family level to parents, rather than primarily to mothers. At times, it may also be appropriate to provide support and education about infant feeding to fathers independently of mothers [35, 36].

In terms of study limitations, it is worth noting, that all three interviewers were female in a study whose purpose is to understand the views of men. Fathers in the study may have expressed themselves differently with male interviewers. Additionally, while one off interviews worked well, the use focus groups with groups of similar fathers would be a valuable addition to future research.

## Conclusion

Fathers in our study valued breastfeeding because it is seen as healthy and natural for their children. Their views on breastfeeding are shaped by their own experiences and observations. Many of the fathers in our study had seen their partners struggle with breastfeeding. As a result, some viewed breastfeeding as a potentially harmful practice for mothers. Their accounts demonstrated that breastfeeding problems affect families, not just mothers and infants. There is scope for improvement in the care of women during and after birth to reduce breastfeeding problems and for fathers to learn more about breastfeeding prior to the birth of their child.

## Additional file

Additional file 1: Interview guide. (DOCX 15 kb)

## Abbreviations

EBM: Expressed breast milk
WHO: World Health Organization
