# Supplementary material for: ‘They’re born to get breastfed’- how fathers view breastfeeding: a mixed method study
Source: BMC Pregnancy Childbirth. 2018 Jun 18;18:238. doi: 10.1186/s12884-018-1827-9 (PMC6006837; doi:10.1186/s12884-018-1827-9)
Supplement: Supplementary file 1 — Interview guide. (DOCX 15 kb) [file 12884_2018_1827_MOESM1_ESM.docx]

**Interview Guide**

**Interviews with Mothers and Fathers/Male/Caregivers and community workers who work with young mothers and fathers**

These are in-depth interviews and each interview will vary a little form the others. Below are listed the types of topics that we would like to discuss in the interviews.

1. Tell me a bit about yourself (yourselves) and your baby (s)?
2. Tell me about your experiences of feeding your baby (s)?
3. (Prompts if needed): How are you currently feeding your baby?
   1. Has this changed over time? Are you happy with the way you are feeding your baby?
   2. What have you liked the most about feeding your baby?
   3. Have you had any problems feeding your baby? (what have you enjoyed / not enjoyed? How they were /may have resolved these?
4. What things do each of you do to help feed your baby (this is a question that explores roles)
5. What or who has helped or influenced you when making feeding decisions?
6. What has helped you to feed your baby the way you wanted to? What did not help you to feed your baby the way you wanted to?
7. Where have you gone for help or support if you needed it? (people, books, websites, other?).
8. Have you had different opinions at times about feeding your baby (s)?
9. In relation to breastfeeding decisions what issues seem to you be the most important when deciding to breastfeed/continue to breastfeed?
10. How can fathers/male caregivers best support mothers when they are feeding babies? (breastfeeding? Bottle feeding? Other? Examples?)
11. How can health professionals (GPs, midwives, child health nurses, others?) improve what they do in relation to infant feeding? (male female specific)
12. Have you got any advice you would like to give to other parents about feeding babies based on your own experiences?
13. Is there Anything else you would like to offer?

**END-** thank you for your time
